# Targeting the foramen ovale is the point of percutaneous balloon compression? Comment on: Robot-assisted percutaneous balloon compression for trigeminal neuralgia-preliminary experiences

**DOI:** 10.1186/s12883-024-03576-5

**Published:** 2024-02-27

**Authors:** Jun Zhong

**Affiliations:** Department Neurosurgery, XinHua Hospital, Shanghai JiaoTong University School of Medicine, 1665 KongJiang Rd, Shanghai, 200092 China

The paper titled “Robot-assisted percutaneous balloon compression for trigeminal neuralgia- preliminary experiences” published in your journal [Li N, et al. BMC Neurol 2023: 22;23(1):163] [1] has prompted me to offer commentary on the integration of modern navigation systems in the process of percutaneous balloon compression (PBC).

The use of robot-assisted techniques may improve cannula engagement with the foramen ovale, but it does not guarantee successful balloon insertion into Meckel’s cave. In fact, precise inflation of the balloon within the cave plays a pivotal role in the overall process [2]. Basically, with a comprehensive understanding of the local anatomy, accessing the foramen ovale under standard lateral fluoroscopy poses no significant challenges. The appealing simplicity of PBC suggests that it is advisable to refrain from unnecessarily complicating a process that is already straightforward.

The following tips may be helpful instead. It is recommended to gently puncture the wall of Meckel’s cave using a fine stylet before introducing the balloon catheter. While advancing the catheter, make sure you recognize damping as an indicative sign of its presence within the cave. Afterwards, a preliminary inflation test is necessary to visually inspect the X-ray image for a pear-shaped sign as an additional verification. Finally, reposition the focus on compressing the semilunar ganglion located at the base of the pear (Table 1).

**Table 1:** A recommended operative procedure of PBC

Steps	Actions	Interpretations
1	Approach	A good angle reaching foramen ovale
2	Puncture	The wall of Meckel’s cave pierced with a fine stylet
3	Penetrate	Catheter advanced in the cave with damping feedback
4	Pear	A pear-shaped head appears with a test inflation
5	Compression	Ganglion at the bottom of the pear in focus

In summary, machinery has limited reliability. With an ultimate comprehension of the spatial existence of Meckel’s cave through observed projection and perceived resistance feedback, one can effortlessly accomplish the PBC process.

## Data Availability

Not applicable.

## References

[1] Li N, Sun T, Hu B, Zhao K, Zhang C, Liu J, Yang C (2023). Robot-assisted percutaneous balloon compression for trigeminal neuralgia- preliminary experiences. BMC Neurol.

[2] Hu S, Huang Z, Wang H, Chen K, Xia L, Dou N, Zhong J (2023). The value of a headless pear shape in Percutaneous Balloon Compression for Trigeminal Neuralgia. Oper Neurosurg (Hagerstown).

